# Correlation between religion, spirituality and perception of healthcare services utilisation in Poland during COVID-19 pandemic

**DOI:** 10.7717/peerj.14376

**Published:** 2022-11-29

**Authors:** Magdalena Tuczyńska, Maja Matthews-Kozanecka, Ewa Baum

**Affiliations:** 1 SSC of Maxillofacial Orthopaedics and Orthodontics, Poznan University of Medical Sciences, Poznan, Greater Poland, Poland; 2 Department of Social Sciences and the Humanities, Poznan University of Medical Sciences, Poznan, Greater Poland, Poland; 3 Division of Philosophy of Medicine and Bioethics, Poznan University of Medical Sciences, Poznan, Greater Poland, Poland

**Keywords:** COVID-19, Religion, Spirituality, Healthcare, Utilisation, Accessibility, Quality

## Abstract

**Background:**

The worldwide transmission of SARS CoV-2 caused the COVID-19 pandemic and had an impact on healthcare provision. The disruption of reliance on the health system during the COVID-19 pandemic posed a clear threat to public trust. Religiosity, like spirituality, is believed to have a positive influence on people’s lives, enabling them to cope with illness, stress, and sudden life changes. In practice, although the terms religiosity and spirituality have similar meanings and are related, they are not identical concepts. The aim of this study is to compare the perceptions of the accessibility and quality of healthcare services provided before and during the COVID-19 pandemic in Poland by religious/spiritual people compared to those for whom religion and spirituality have little or no importance in their lives.

**Methodology:**

This cross-sectional study was based on the authors’ questionnaire, carried out during the third wave of the COVID-19 pandemic in Poland. Participants in the study were people living in various regions of Poland over 18 who were willing to complete the questionnaire voluntarily. The number of people sampled was two hundred and sixty-four. Convenience sampling method was used for this study. Statistical calculations were performed using Statistica 13 software from TIBCO and PQStat from PQStat Software and were based on the Kruskal-Wallis test, multiple regression model, the chi2 test of independence or the Mann-Whitney test. The result was considered statistically significant when *p* < *α*. The significance level was taken as *α* = 0.05.

**Results:**

Two hundred and sixty-three respondents answered the questionnaire. Among them, 181 (69%) were women, and 82 (31%) were men. It was shown that religion is more important for women than for men and women who report a high role of religion in their lives rated the quality of healthcare services better before and during the COVID-19 pandemic. It was also concluded that religious people for whom religion played a major role and those who were helped by spiritual life rated accessibility and quality higher both before and during the pandemic.

**Conclusions:**

Religious/spiritual people, through their more positive attitudes towards the world, were thought to rate access and quality of healthcare services better. Regardless of religious affiliation, the help of spiritual life during the pandemic or the importance of religion in life in all respondents, perception of healthcare services utilisation were decreased by the pandemic. This prompts thoughts on the implementation of spiritual assistance as a supportive measure to mitigate the effects of the pandemic.

## Introduction

Religion and spirituality positively impact many psychosocial and health factors throughout life. Most people use religion or spirituality to manage illness or stress, and there is evidence that this also improves mental and physical health, at least in a clinical context (VanderWeele, 2017). The terms religiosity and spirituality are interrelated, but in contemporary practice, it is essential to differentiate between them. It is believed that religion is usually more organised and institutionalised than spirituality and, as such, provides more opportunities for social interaction and social benefits. Nevertheless, spirituality generally lacks the institutionalised element of religion but is not considered a separate construct. By definition, religion means beliefs, practices, and rituals related to transcendence, where the transcendent is God, Allah, Buddha, a Higher Power, *etc*. Religions usually have specific beliefs about life after death and principles of behaviour in a social group. Religion is associated with traditional values and practices. A religious person is linked to a particular faith, God, sacred writings, values, and ethics. Spirituality is intimately connected to the supernatural, the mystical, and organised religion. Spirituality includes both a search for the transcendent and the discovery of the transcendent (Koenig, 2012; Van Cappellen et al., 2016; Fowler, 2017; Lavorato Neto et al., 2018).

According to World Health Organization, at the beginning of March 2020, due to the novel coronavirus, now known as Severe Acute Respiratory Syndrome Coronavirus-2 (SARS-CoV-2), there was an outbreak of the COVID-19 pandemic. The spread of the SARS-CoV-2 virus has caused thousands of deaths and limitations in access to healthcare, diagnostics, and personal protective equipment (Baloch et al., 2020; Ciotti et al., 2020; Kaye et al., 2020). Although the transmission of the coronavirus was delayed in Poland compared to some other countries, on 24 March, the Polish Ministry of Health introduced self-isolation measures to limit the spread of the disease (Szczepańska & Pietrzyka, 2021; Walkowiak & Walkowiak, 2022). Countries have tried to implement various solutions to limit the spread of the SARS-CoV2 virus. Restrictions have covered lockdowns, the obligation of personal protective equipment in public spaces, restriction of visiting patients in healthcare facilities, the limit of people in shops, social distance, and many others (Studdert & Hall, 2020; Hugelius, Harada & Marutani, 2021).

During the COVID-19 pandemic, there was a disruption in reliance on the healthcare system. Reduced regular healthcare options, increasing medical uncertainty, decision-making conflicts, and changing treatment algorithms in a pandemic posed explicit threats to public trust in the healthcare system (Beller et al., 2022). The SARS-CoV2 pandemic caused a global decline in healthcare utilisation. People with diseases other than COVID-19 declared reduced access to healthcare services (Moynihan et al., 2021). Several factors influence the perception of public health services. These factors vary considerably from country to country (Zhao, Zhao & Cleary, 2019).

It is considered that about 90% of the world’s population is involved in some religious/spiritual practice, and this dimension plays a vital role in life. Religious affiliation influences daily life and health-related decisions. Scientific findings provide information showing that religion is relevant not only in daily life but also in health-related decisions and attitudes. Additionally, how patients experience the religious/spiritual dimension can be protective or harmful, depending on how these resources are used. Differentiation based on religion and spirituality was found in terms of physical and mental health and the use of healthcare services. The results of various studies indicate that people who regularly attend religious services trust physicians and the healthcare system more than those who attend less often or never (Benjamins, 2006; Moreira-Almeida, Neto & Koenig, 2006; Borges et al., 2021).

Several international studies have validated the impact of the COVID-19 pandemic on risks to mental well-being. The pandemic’s daily life has also contributed to a decline in mood among Poles (Stańdo et al., 2022). Spirituality and religion can be a positive reassurance in societies facing epidemics and a help in times of crisis (Fardin, 2020). During the distressing crisis of the COVID-19 pandemic, where the population was faced with restrictions, lockdowns, and limitations on access to a variety of services, many people turned to religion for psychological relief (Tan, Musa & Su, 2022). The study conducted in Brazil showed that spirituality protected against chronic and acute anxiety (Tolentino et al., 2022) and the one in Czech, that both religiosity and spirituality emphasised positive changes in some areas of behaviour and feelings during the pandemic, such as feelings of helplessness and the lack of spirituality increased helplessness, fear, and anxiety (Buchtova et al., 2022).

Although there are numerous studies on the impact of spirituality and religion on health during the COVID-19 pandemic, in terms of healthcare service utilisation, it represents a niche level worth filling. This study aims to compare the perception of the accessibility and quality of healthcare services provided before and during the COVID-19 pandemic in Poland among religious/spiritual people *versus* those for whom religion and spirituality have little or no importance in their lives. We believe this study is relevant because it shows how spirituality and inner resources can help during a challenging period such as a pandemic and that spiritual support has the potential to reverse perceptions of healthcare utilisation.

## Materials & Methods

### Study design

This cross-sectional study was based on the authors’ questionnaire and carried out during the third wave of the COVID-19 pandemic in Poland between 8 July and 11 August in 2021. The Bioethics Committee approved it at Poznan University of Medical Sciences (Institutional Review Board number 484/21) in conformity with the Helsinki Declaration guidelines. The questionnaire was designed by the authors especially for this study. The questions were prepared in Polish, which is the native language of the respondents. The questions in the questionnaire focused on accessibility and quality of healthcare services during the COVID-19 pandemic. The questionnaire contained 22 questions, 12 of which focused on the sociodemographic characteristics of respondents and 10 on the accessibility and quality of healthcare services during the COVID-19 pandemic in Poland. These questions included religious belonging, the importance of religion in life, and whether spiritual life helped during the pandemic. Also, questions using the VAS scale to assess the accessibility and quality of healthcare services before and during the COVID-19 pandemic from 1 to 10, where 1 meant very bad and 10 meant very good. The Visual Analogue Scale is a method for measuring subjective characteristics or attitudes that cannot be directly assessed.

### Participants

The number of people sampled was two hundred and sixty-four, living in various regions of Poland over 18 who were willing to complete the questionnaire voluntarily. Convenience sampling method was used for this study. Exclusion criteria for the study included patients living outside Poland, minors, and those who did not consent to participate in the study. On 8 July 2021, an invitation was distributed *via* the Google form with a link to the questionnaire, which was shared on social media. The questionnaire was also distributed in paper to Poznan University of Medical Sciences patients. Respondents were explained the purpose of the questionnaire and informed that participation was voluntary and confidentiality would be maintained. Written consent was also obtained.

### Statistical analysis

The analysis of the data was based on calculations made with statistical tools—Statistica 13 software from TIBCO and PQStat from PQStat Software. It was defined that statistically significant results are those where *p* < *α* and the significance level was considered to be *α* = 0.05. Statistical analysis of scale reliability and Cronbach’s alpha coefficient, verifying the validity and reliability of the questionnaire, demonstrated that all questions from the questionnaire could remain. The Kruskal-Wallis test was calculated to compare variables. The chi2 test of independence or the Mann–Whitney test was calculated to examine correlations. A multiple regression model was used to compare the dependent variable, *i.e.,* the assessment of accessibility and quality of healthcare services, and the two independent variables.

## Results

### Characteristics of the participants

Two hundred and sixty-four respondents answered the questionnaire. Among them, 181 (69%) were women, and 82 (31%) were men, one person did not declare gender. One hundred forty-nine respondents declared religious affiliation, 47 declared no religious affiliation, and 67 people did not answer this question. The question of the meaning of religion in life was responded to by 254, and the importance of spirituality by 261 people. The specific variables regarding the characteristics of the respondents are presented in Table 1.

**Table 1:** Characteristics of respondents.

Independent variables	Categories	*N*	%
Sex	Female	181	68,82
	Male	82	31,18
Affilation	Declare religious affiliation	149	76,02
	Declare no religious affiliation	47	23,98
Meaning of religion in life	Does not play a role	57	22,44
		Plays a minor	41	16,14
		Difficult to say	72	28,35
		Plays a major role	63	24,80
	Has a key role	21	8,27
Meaning of spiritual life during COVID-19 pandemic	Helpful	139	53,26
	Not helpful	122	46,74

### Gender correlation

Based on Pearson’s Chi $\hat {}$2 test, there was no correlation between spiritual life and gender (*p* = 0.480) and religious affiliation and gender (*p* = 0.108). A correlation was found between the importance of religion in life and gender (*p* = 0.004), which indicates that for women, religion is more important in life than for men. Since a correlation was shown between gender and the importance of religion in life, a statistical revision of the original analysis was performed based on a multiple regression model with the dependent variable being the accessibility/quality score and the independent variables being gender and the importance of religion in life. Assessing accessibility to healthcare services before the COVID-19 pandemic against the importance of religion in life by gender showed significance in a multiple regression model (*p* = 0.000845) and that after including gender, ‘plays a big role’ is still significant when assessing accessibility to healthcare services before the pandemic (*p* = 0.000086). In contrast, during the COVID-19 pandemic, the model was not significant (*p* = 0.066412). Regarding the assessment of the quality of healthcare services in relation to the importance of religion in life by gender both before the pandemic (*p* = 0.023053) and during (*p* = 0.037747) the pandemic, the multiple regression model was significant. Also, considering gender, ‘plays a big role’ was significant when assessing quality before (*p* = 0.002894) and during (*p* = 0.001423) the COVID-19 pandemic.

### Comparison of the assessment of access to healthcare services before and during a COVID-19 pandemic

**Figure 1:**
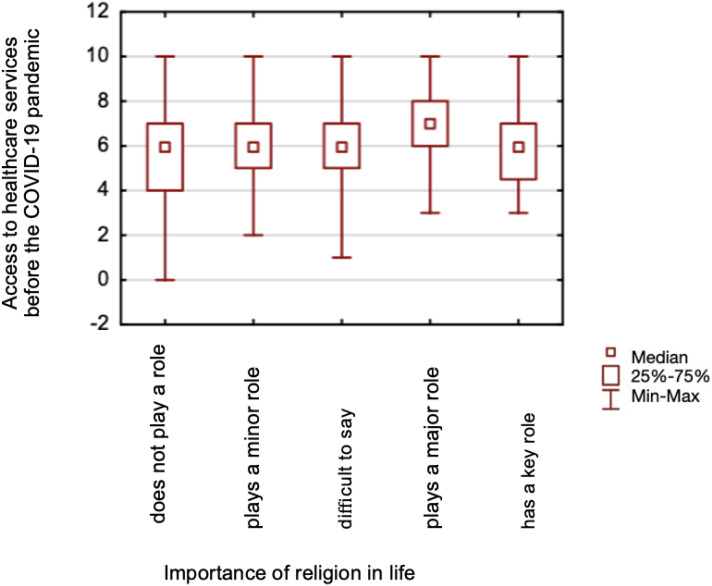
A comparison of healthcare services accessibility assessment before the COVID-19 pandemic, and the importance of religion in life.

**Figure 2:**
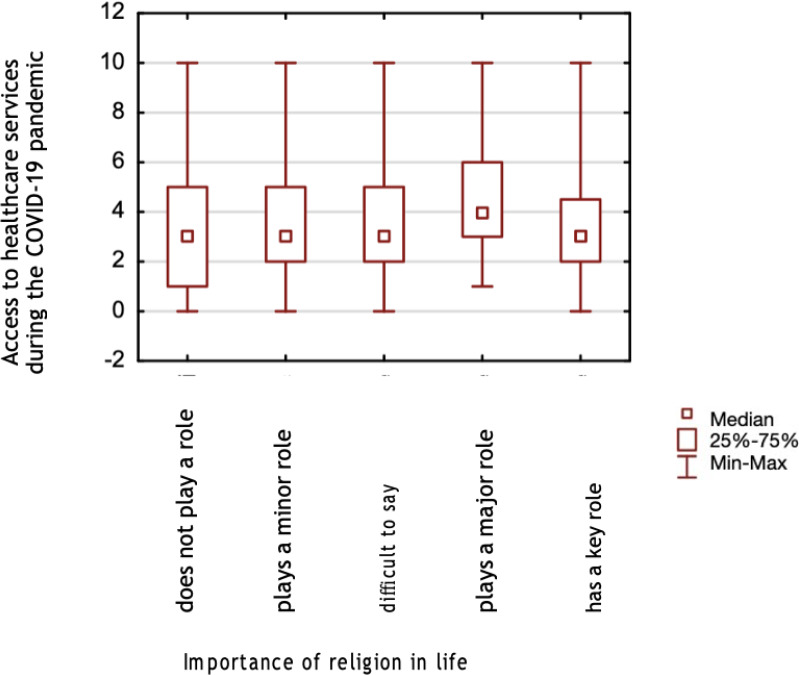
A comparison of healthcare services accessibility assessment during the COVID-19 pandemic, and the importance of religion in life.

The study found no differences in assessing accessibility to healthcare services before (*p* = 0.130) and during (*p* = 0.146) the pandemic between those declaring a religious affiliation and those declaring no religious affiliation. No differences also were found when comparing respondents that claimed that spirituality helped them during the COVID-19 pandemic and those who had not been helped by spirituality, for both pre-pandemic (*p* = 0.135) and during (*p* = 0.084). Mann–Whitney test was calculated for both analyses. Based on the Kruskal-Wallis test, significant differences were found in the assessment of accessibility to healthcare services before the pandemic (*p* = 0.0004) between those for whom religion plays a major role in life and those for whom it does not play a role, its difficult for them to say and plays a minor role (Fig. 1). Significant differences were also found in assessing accessibility during the pandemic (*p* = 0.0144) between those for whom religious life plays a major role and those for whom it does not at all (Fig. 2).

### Comparison of the assessment of the quality of healthcare services before and during a COVID-19 pandemic

**Figure 3:**
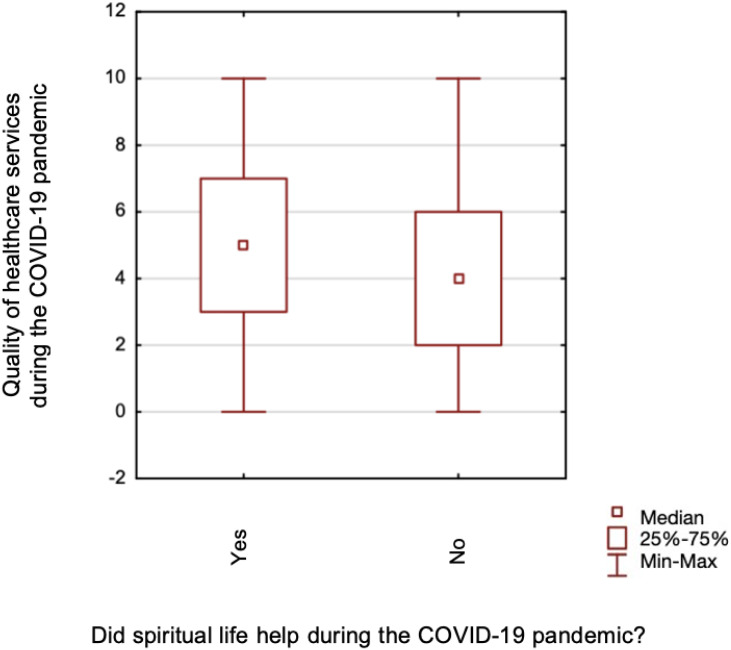
A comparison of healthcare services quality assessment during the COVID-19 pandemic, and spiritual life support during the pandemic.

**Figure 4:**
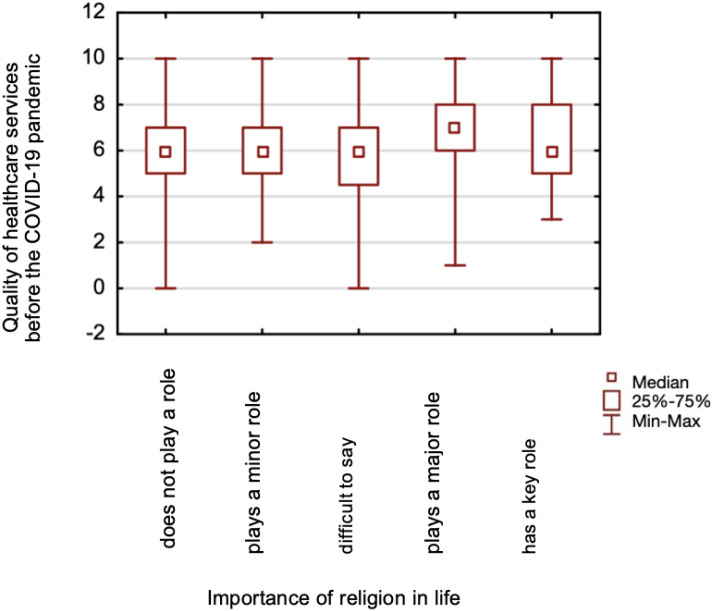
A comparison of healthcare services quality assessment before the COVID-19 pandemic, and the importance of religion in life.

**Figure 5:**
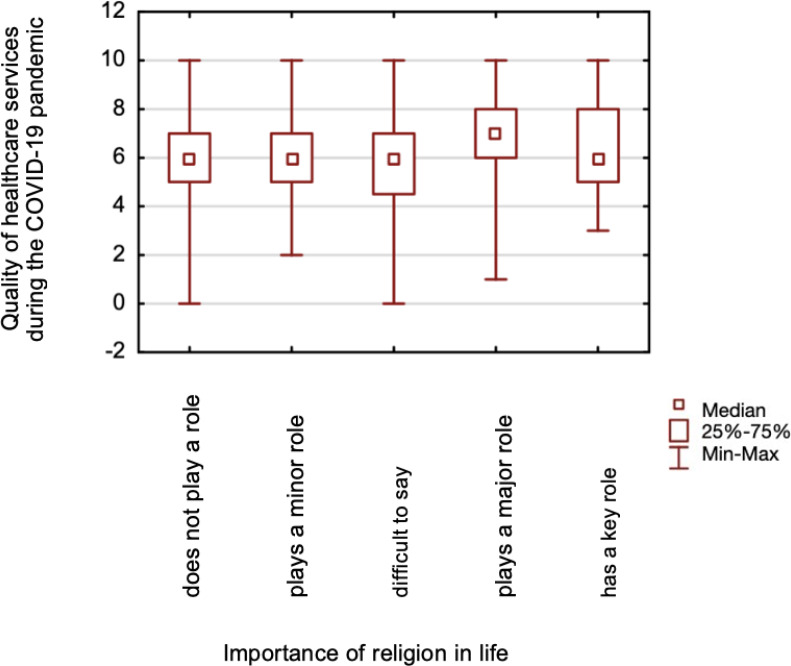
A comparison of healthcare services quality assessment during the COVID-19 pandemic, and the importance of religion in life.

The study found no differences in assessing the quality of healthcare services before (*p* = 0.137) and during (*p* = 0.161) the pandemic between those declaring a religious affiliation and those claiming no religious affiliation. No differences were found when comparing those respondents whose spiritual life helped during the pandemic and those who did not help during the pre-pandemic period (*p* = 0.199). Still, there were statistically significant differences in the quality of healthcare services assessment during the COVID-19 pandemic (*p* = 0.038) (Fig. 3). Mann–Whitney test was calculated for the above analyses. Based on the Kruskal-Wallis test, significant differences were found in the assessment of the quality of healthcare services before the pandemic (*p* = 0.025) (Fig. 4), and during (*p* = 0.024) (Fig. 5), between those for whom religion plays a major role in life and those for whom it does not play a role at all.

### Assessment of the impact of the COVID-19 pandemic on the accessibility and quality of healthcare services

To determine whether the assessment of the accessibility and quality of healthcare services was affected by the pandemic, the Wilcoxon Matched-Pairs Signed Ranks Test was performed. Statistically significant differences (*p* = 0.000) were found in all variables examined, *i.e.,* for those with religious affiliation and those without, for those whose spiritual life helped during the pandemic and those who did not, and for those for whom religion is not important, is of minor importance, is difficult for them to say, plays a major role and is of key importance (Table 2).

**Table 2:** Table showing the change in assessment of accessibility and quality to healthcare services in COVID-19 pandemic.

		*n*	Mean score	Standard deviation	*p* value
Religiously affiliated respondents	Accessibility before COVID-19	147	6.35	1.93	0.000
			Accessibility during COVID-19	3.95	2.40	0.000
		Quality before COVID-19	148	6.55	1.94	0.000
	Quality during COVID-19	4.68		2.70	0.000
Non-religiously affiliated respondents	Accessibility before COVID-19	47	5.77	2.23	0.000
			Accessibility during COVID-19		3.45	2.51	0.000
			Quality before COVID-19		5.87	2.44	0.000
	Quality during COVID-19		4.08	2.97	0.000
Respondents whom spiritual life helped during the COVID-19 pandemic	Accessibility before COVID-19	136	6.30	2.07	0.000
			Accessibility during COVID-19	4.07	2.61	0.000
		Quality before COVID-19	137	6.44	2.02	0.000
	Quality during COVID-19	4.76		2.87	0.000
Respondents whom spiritual life did not helped during the COVID-19 pandemic	Accessibility before COVID-19	121	5.82	2.15	0.000
			Accessibility during COVID-19	3.45	2.25	0.000
		Quality before COVID-19	120	5.99	2.27	0.000
	Quality during COVID-19	4.02		2.52	0.000
Importance of religion in life: does not play a role	Accessibility before COVID-19	57	5.53	2.36	0.000
			Accessibility during COVID-19		3.19	2.50	0.000
			Quality before COVID-19		5.70	2.42	0.000
	Quality during COVID-19		3.60	2.89	0.000
Importance of religion in life: has a minor role	Accessibility before COVID-19	40	5.70	2.01	0.000
			Accessibility during COVID-19		3.50	2.37	0.000
			Quality before COVID-19		5.85	2.21	0.000
	Quality during COVID-19		4.27	2.70	0.000
Importance of religion in life: is difficult to say	Accessibility before COVID-19	72	5.83	1.99	0.000
			Accessibility during COVID-19		3.69	2.45	0.000
			Quality before COVID-19		6.07	1.94	0.000
	Quality during COVID-19		4.24	2.50	0.000
Importance of religion in life: plays a major role	Accessibility before COVID-19	61	7.07	1.79	0.000
			Accessibility during COVID-19	4.56	2.25	0.000
		Quality before COVID-19	62	6.92	1.92	0.000
	Quality during COVID-19	5.24		2.73	0.000
Importance of religion in life: has a key role	Accessibility before COVID-19	20	5.95	2.01	0.000
			Accessibility during COVID-19		3.65	2.56	0.000
			Quality before COVID-19		6.45	2.19	0.000
	Quality during COVID-19		4.50	2.74	0.000

For religiously affiliated respondents, the mean score for accessibility to healthcare services before the COVID-19 pandemic was 6.35 and during was 3.95, while the mean quality score was 6.55 before the pandemic and 4.68 during. In comparison, for non-religiously affiliated respondents, the mean score for accessibility to healthcare services before the pandemic was 5.77 and during was 3.45, while the mean quality score was 5.87 before the pandemic and 4.08 during. For respondents who felt that spiritual life helped during the COVID-19 pandemic, the mean score for accessibility to healthcare services before the COVID-19 pandemic was 6.30 and during was 4.07, while the mean quality score was 6.44 before the pandemic and 4.76 during. In comparison, for those who felt that spiritual life did not help them during the pandemic, the mean score for accessibility to healthcare services before the pandemic was 5.81 and during was 3.45, and the mean quality score was 5.99 before the pandemic and 4.02 during.

## Discussion

This study aimed to compare the perception of the accessibility and quality of healthcare services provided before and during the COVID-19 pandemic in Poland among religious/spiritual people *versus* those for whom religion and spirituality have little or no importance in their lives. Firstly, the study indicates that for women, religion is more significant in their lives than for men. This is in line with other scientific reports, which state that women are indeed generally more religious than men. There is some evidence from the studies that the association between spirituality/religiosity and health is not the same across genders (Maselko & Kubzansky, 2006). This follows from risk preference theory, as women tend to avoid risk more than men, so religion is more important to them in their lives (Li et al., 2020). In addition, gender differences in religiosity are considered to be contributed by country-level gender equality (Moon, Tratner & McDonald, 2022). In relation to the COVID-19 pandemic, studies have shown that women were more likely than men to attend mass, say prayers, and were more enthusiastic about national leadership and local churches (Meza, 2020; Francis & Village, 2022). Interestingly, a study conducted among Poles during the COVID-19 pandemic showed no statistical significance with regard to the correlation between gender and religious commitment (Boguszewski, Makowska & Podkowińska, 2022). In addition, statistical analysis showed that women for whom religion is important rated the quality of healthcare services higher before and during the COVID-19 pandemic. Still, access was rated higher only before the pandemic and not during it. The fact that women did not rank access to healthcare services higher during the COVID-19 pandemic is interesting and requires further research.

Healthcare accessibility is the relative comfort of receiving healthcare services in a particular place (Ma et al., 2018). In addition to the accessibility of healthcare services, its quality also plays an imperative role. Even with decent access and quality of healthcare services, some barriers pose limitations, among them financial, social, organisational, and cultural factors (Lee & Kim, 2017; Núñez, Sreeganga & Ramaprasad, 2021). We found that the assessment of accessibility, as well as the quality of healthcare services both before and during the pandemic, was higher for people for whom religion is of major importance in their lives than for people for whom it is of minor importance. In addition, an analysis of the assessment of the impact of the COVID-19 pandemic on accessibility and quality of healthcare services showed that those who were more religious/spiritual rated both accessibility and quality of healthcare services higher during the pandemic than those who did not declare a religious affiliation and those whose spirituality did not help during the pandemic. Such results may be because religious/spiritual people are more open to others, more trusting, view the world more positively, and evaluate situations more favourably (Khoynezhad, Rajaei & Sarvarazemy, 2012; Berthold & Ruch, 2014; Lyon et al., 2016; Baum, 2017). Religion, in association with science and government, was an important component in combating the COVID-19 pandemic (Lee et al., 2022). A study among Polish society on religion and faith perception during COVID-19 has found that most of the respondents declared that spirituality was important to them in the face of Covid-19 pandemic and the protective power of faith and the sense of security increased (Kowalczyk et al., 2020). Another study among UK and US citizens found that, during the COVID-19 pandemic, strongly religious believers increased their commitment to religious beliefs, while those for whom faith has a minor significance reported an increase in scepticism towards religion (Rigoli, 2021). However, it should be mentioned that regardless of the declared religious affiliation, the importance of religion in life, or the declaration that spiritual life helped during the pandemic, in all groups, it was shown in our study that both the perception of access and quality of healthcare services decreased because of COVID-19 outbreak.

Access and quality of healthcare services were limited due to the COVID-19 pandemic, with a decline affecting almost every country in the world (Topriceanu et al., 2021; Howarth et al., 2021). This was due to an increase in the number of patients admitted to hospital wards and a shortage of medical staff or personal protective equipment (Babroudi, Sabri-Laghaie & Ghoushchi, 2021). In Poland, access to healthcare services was already limited before the pandemic, and the SARS-CoV2 outbreak only exacerbated the problem (Zienkiewicz, Zienkiewicz & Dziaduch, 2018; Kasiukiewicz & Wojszel, 2021). Both chronically ill patients and those requiring emergency care have been affected (Nadolny et al., 2021; Grudziąż-Sękowska, Sękowski & Kobuszewski, 2022). Research in health psychology has shown that spirituality positively impacts people. It could benefit the healthcare system to educate medical personnel who would provide spiritual support to patients. Appropriate education about spiritual needs in Polish medical institutions could improve the quality of care in Polish hospitals (Klimasiński et al., 2022). And in terms of the potential benefits of religion during a pandemic, it is thought that high religious engagement may mitigate the harmful effects of tragedy and stressful events. It also correlates with reduced disease progression, as well as mortality rates (Knight, Dudenkov & Cheshire, 2021).

Patients’ perceptions of healthcare utilization are significantly related to empathy, trust and perceived daily discrimination (Torain et al., 2021), additionally patients’ insights are key to improving the utilisation of the healthcare system worldwide and can provide relevant and comprehensive information (Al-Jabri, Turunen & Kvist, 2021). At the time of the great challenge of the COVID-19 pandemic, the authors wanted to investigate the importance of the correlation between religion, spirituality and the perception of healthcare service utilisation due to the fact that the majority of the Polish population is of religious/spiritual people, and so it seemed likely that such people could significantly better evaluate the healthcare services. Religion plays an crucial role in the lives of Poles. Although the statement of attitudes to religious belief shows only a rough picture of the importance and role of religion in people’s lives, it is one of the most common measures of religiosity. Nearly 94% of the Polish population aged 16 and over declare a religious affiliation with almost 92% declare their affiliation with the Roman Catholic Church and almost 81% consider themselves to be religious believers (Central Statistical Office, 2018). Spirituality and internal resources can help during a difficult time like a pandemic. Spiritual and religious patients are thought to rate clinical care more positively (Williams et al., 2011), and although there are studies that have examined this issue before, the authors wanted to present the concept because of the religious homogeneity shared values and close spirituality among the Polish population.

### Limitations and future research

The study shows the importance of religion and spiritual life on the perception of public healthcare services. Nevertheless, the conducted research has its limitations, namely: data collected during only one pandemic wave, lack of specific differentiation between direct and teleconsultation visits, the lack of representativeness, collecting responses both on paper and online, the exclusion of pediatric patients and inability to reach selected groups of patients, *e.g.*, in nursing homes or palliative care. Further research should distinguish between healthcare services provided in person by physicians and those provided *via* telemedicine. Additionally, the questionnaire among respondents from another European country would be beneficial for comparison.

## Conclusions

The impact of the COVID-19 pandemic has affected various aspects of life in almost every country in the world. The field of public health was particularly acutely affected. Hospitals were overcrowded, personal protective equipment was in short supply, and medical staff were insufficient. All this affected access to and quality of healthcare services. Studies conducted worldwide have drawn attention to the decline in the quality and accessibility of these services. This situation has also occurred in Poland. As religiosity and spiritual life are one of the main personal resources favouring the appearance of positive changes in people, it could be hypothesised that religious/spiritual people will evaluate the utilisation of healthcare services more positively. The analysis of the results of our study confirmed this hypothesis. However, it would be worthwhile to continue the study for further conclusions.

##  Supplemental Information

10.7717/peerj.14376/supp-1 Supplemental Information 1 Anonymised raw data These data were used to provide an analysis of the correlation between religion, spirituality and the use of healthcare services in Poland during the COVID-19 pandemic.

10.7717/peerj.14376/supp-2 Supplemental Information 2 Questionnaire

10.7717/peerj.14376/supp-3 Supplemental Information 3 Questionnaire in orginal language

## Additional Information and Declarations

### Data Availability

The following information was supplied regarding data availability: The raw measurements are available in the tables.
