# Supplementary material for: Correlation between religion, spirituality and perception of healthcare services utilisation in Poland during COVID-19 pandemic
Source: PeerJ. 2022 Nov 29;10:e14376. doi: 10.7717/peerj.14376 (PMC9744140; doi:10.7717/peerj.14376)
Supplement: Supplemental Information 3 [file peerj-10-14376-s003.pdf]

Kwestionariusz ankiety dotyczący dostępności do świadczeń zdrowotnych w okresie pandemii COVID-19

SZANOWNY PANIE, SZANOWNA PANI !

Uprzejmie proszę o wzięcie udziału w badaniu i wypełnienie kwestionariusza. Celem tego badania jest poznanie opinii pełnoletnich Pacjentów na temat dostępności do świadczeń zdrowotnych w okresie pandemii COVID-19. Badanie ma charakter anonimowy. Po wypełnieniu kwestionariusza nie należy składać podpisu. Wnioski sformułowane w wyniku zbiorczego opracowania ankiety zostaną wykorzystane do opracowań naukowych. Przed wypełnieniem kwestionariusza proszę o uważne zapoznanie się z jego treścią. Udzielając odpowiedzi na kolejne pytania, należy spośród podanych odpowiedzi wybrać odpowiedź(i) zgodną(e) z osobistymi poglądami lub wpisać odpowiedź we właściwym miejscu. Proszę o udzielenie odpowiedzi na każde pytanie.

Wypełnienie ankiety jest równoznaczne z wyrażeniem zgody na udział w badaniu.

Serdecznie dziękuję za uważne i szczere wypełnienie ankiety.

Magdalena Tuczyńska

METRYCZKA:
pole wybranej odpowiedzi proszę zaznaczyć - X

KOBIETA - ❑ MĘŻCZYZNA - ❑

**Jakie jest Pana/Pani miejsce zamieszkania:**<br><br>• **miasto do 50 tys. mieszkańców ❑**<br>• **miasto powyżej 50 tys. do 100 tys. mieszkańców ❑**<br>• **miasto powyżej 100 tys. mieszkańców ❑**<br>• **wieś ❑**<br><br>**Czy mieszka Pan/Pani:**<br><br>• **samotnie ❑**<br>• **z żoną/mężem ❑**<br>• **z partnerką/partnerem ❑**<br>• **z rodziną ❑**<br>• **inne ❑**	**Ile ma Pan/Pani lat:**<br>**mężczyźni 18-64 lata ❑**<br>**mężczyźni powyżej 65 lat ❑**<br>**kobiety 18-59 lat ❑**<br>**kobiety powyżej 60 lat ❑**<br><br>**Czy jest Pan/Pani osobą z określoną niepełną sprawnością:**<br>**tak ❑**<br>**nie ❑**

**<u>Jaka jest Pana/Pani sytuacja:</u>**	**<u>Jakie ma Pan/Pani wykształcenie:</u>**
• **utrzymuje się samodzielnie ❑**<br>• **jestem na utrzymaniu rodziców, innych osób ❑**<br>• **częściowo jestem na utrzymaniu rodziców, innych osób ❑**<br><br>**<u>Ile wynosi osiągany przez Pana/Panią miesięczny dochód:</u>**<br><br>• **poniżej średniej krajowej ❑**<br>• **równy średniej krajowej ❑**<br>• **powyżej średniej krajowej ❑**<br>• **nie osiągam dochodów ❑**	• **podstawowe ❑**<br>• **gimnazjalne ❑**<br>• **ponadgimnazjalne ❑**<br>• **policealne ❑**<br>• **<u>wyższe:</u>**<br>**licencjackie ❑**<br>**magisterskie ❑**<br>**doktorat i powyżej ❑**

**<u>Proszę określić Pana/Pani samoocenę stanu zdrowia:</u>**	**<u>Jaka jest Pana/Pani przynależność religijna:</u>**
• **bardzo dobre ❑**<br>• **dobre ❑**<br>• **takie sobie, ani dobre ani złe ❑**<br>• **złe ❑**<br>• **bardzo złe ❑**	**…………………………………………………………**<br><br>**<u>Jakie znaczenie ma dla Pana/Pani religia w życiu:</u>**<br><br>• **ma kluczowe znaczenie ❑**<br>• **odgrywa dużą rolę ❑**<br>• **trudno powiedzieć ❑**

1.Czy w okresie pandemii COVID-19 korzystał/a Pan/Pani z świadczeń medycznych?:

❑ tak
❑ nie

Jeżeli tak, to proszę zaznaczyć z jakich (można zaznaczyć więcej niż jedną odpowiedź):

❑ świadczenia w ramach powszechnego ubezpieczenia zdrowotnego (NFZ)
❑ świadczenia pełnopłatne
❑ świadczenia w ramach Podstawowej Opieki Zdrowotnej (POZ)
❑ inne, proszę wymienić jakie
………………………………………………………………………………………

2. Czy Pan/Pani zachorował/a na COVID-19?:

❑ tak
❑ nie

Jeżeli tak, to czy był problem z (można zaznaczyć więcej niż jedną odpowiedź):

❑ umówieniem się na wizytę do lekarza POZ
❑ udzieleniem bezpośredniej porady lekarskiej

❑ udzieleniem teleporady
❑ inne, proszę wymienić jakie
……………………………………………………………………………………………

3. Czy w czasie pandemii COVID-19 korzystał/a Pan/Pani ze świadczeń specjalistycznych?:

❑ tak
❑ nie

Jeżeli tak, to proszę zaznaczyć z jakich:

❑ kardiologicznych
❑ internistycznych
❑ laryngologicznych
❑ psychiatrycznych
❑ psychologicznych
❑ dermatologicznych
❑ pulmonologicznych
❑ neurologicznych
❑ ortopedycznych
❑ ginekologiczno - położniczych
❑ urologicznych
❑ endokrynologicznych
❑ okulistycznych
❑ stomatologicznych
❑ pielęgniarskich
❑ fizjoterapeutycznych
❑ inne, proszę wymienić jakie
……………………………………………………………………………………………

4. Czy w czasie trwania pandemii COVID-19 miał/a Pan/Pani problem z otrzymaniem skierowania na badania diagnostyczne?:

❑ tak
❑ nie

Jeżeli tak, to proszę zaznaczyć jakie (można zaznaczyć więcej niż jedną odpowiedź):

❑ RTG
❑ rezonans magnetyczny (MRI)
❑ tomograf komputerowy (TK)
❑ ultrasonografia (USG)
❑ mammografia
❑ badania laboratoryjne
❑ inne, proszę wymienić jakie
……………………………………………………………………………………………

5. Czy w czasie pandemii COVID-19 ktoś bliski z Pana/Pani rodziny lub ze znajomych przebywał na oddziale COVID-19?:

❑ tak
❑ nie

Jeżeli tak, to proszę zaznaczyć, czy była możliwość (można zaznaczyć więcej niż jedną odpowiedź):

❑ odwiedzin
❑ kontaktu telefonicznego
❑ wideorozmowy

❑ przekazania paczki
❑ dostępu do informacji medycznych
❑ inne, proszę wymienić jakie
……………………………………………………………………………………………

6. Czy w czasie pandemii COVID-19 odwołał/a Pan/Pani wizytę lekarską?:

❑ tak
❑ nie

Jeżeli tak, to proszę zaznaczyć przyczynę (można zaznaczyć więcej niż jedną odpowiedź):
❑ obawa przez zakażeniem
❑ kwarantanna
❑ obawa związana z korzystaniem z transportu publicznego
❑ inne, proszę wymienić jakie:
……………………………………………………………………………………………

Jeżeli tak, to proszę zaznaczyć, z jakiej innej formy leczenia, skorzystał/a Pan/Pani (można zaznaczyć więcej niż jedną odpowiedź):
❑ porada farmaceuty w aptece
❑ domowe sposoby lecznicza
❑ porada przez Internet (wyszukiwarka internetowa, fora internetowe)
❑ inne, proszę wymienić jakie:
……………………………………………………………………………………………

7. Czy Pana/Pani zdaniem w czasie pandemii COVID-19 były przestrzegane Prawa Pacjenta?:

❑ tak
❑ nie

8. Czy życie duchowe (uważność, praktyki religijne, medytacja, skupienie, rozwijanie własnych pasji, wsparcie bliskich osób i/lub wykorzystanie innych zasobów wewnętrznych) pomagało Panu/Pani w czasie pandemii COVID-19?:

❑ tak
❑ nie

9. Jak ocenia Pan/Pani dostępność do świadczeń zdrowotnych w trakcie pandemii COVID-19?:

PRZED PANDEMIĄ W TRAKCIE PANDEMII

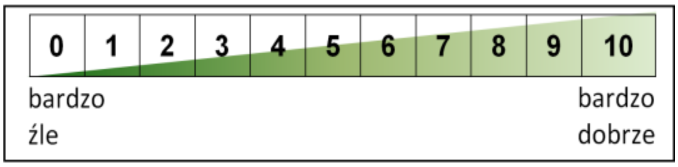

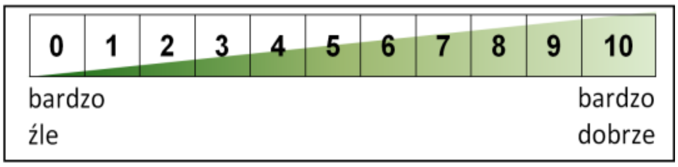

10. Jak ocenia Pan/Pani jakość świadczeń zdrowotnych w trakcie pandemii COVID-19?:

PRZED PANDEMIĄ W TRAKCIE PANDEMII

0	1	2	3	4	5	6	7	8	9	10

bardzo źle — bardzo dobrze

0	1	2	3	4	5	6	7	8	9	10

bardzo źle — bardzo dobrze

Pytania otwarte:

Jeśli Pan/Pani ma jakiekolwiek uwagi dotyczące dostępności do świadczeń zdrowotnych proszę wskazać je poniżej:

………

………
